# Hopes expressed in birth plans by women diagnosed with fetal anomalies: a qualitative study in Japan

**DOI:** 10.1186/s12884-022-05115-x

**Published:** 2022-10-23

**Authors:** Maki Kitazono Chiba, Shigeko Horiuchi, Satomi Ishikawa, Naoko Arimori

**Affiliations:** 1 St. Luke’s International University, 10-1 Akashi-cho, Chuo-ku, Tokyo, 104-0044 Japan; 2 Kanagawa Children’s Medical Center, Nursing Department, 2-138-4 Mutsukawa, Minami-ku, Kanagawa, 232-8555 Japan; 3 Graduate School of Health Sciences, Faculty of Medicine, Niigata University, 2-746 Asahimachi-dori, Chuo-ku, Niigata, 951-8518 Japan

**Keywords:** Birth planning, Family-centered care, Fetal anomalies, Hope, Fetal abnormality, Abnormal fetus, Japanese women

## Abstract

**Background:**

Recent advances in prenatal screening and diagnosis have resulted in an increasing number of women receiving a diagnosis of fetal anomalies. In this study, we aimed to clarify the hopes for childbirth and parenting of women diagnosed with fetal anomalies and to suggest a family-centered care tailored for this situation in perinatal settings.

**Methods:**

A descriptive qualitative study was performed. We recruited women diagnosed with fetal anomalies who were over 22 years old, beyond 22 weeks of gestation, and had scheduled pregnancy and delivery management at a tertiary perinatal medical center specializing in neonatal and pediatric care in a metropolitan area of Japan from April 2019 to December 2019. Women who were willing to participate received support from a midwife to create birth plans. Data were collected from the documented birth plans submitted by 24 women and analyzed using content analysis.

**Results:**

We identified three themes of women’s hopes based on the descriptions of the submitted birth plans: (1) Hopes as women who are expecting childbirth, (2) Hopes as mothers of a baby, (3) Hopes of being involved in the family needs. Several distinctive hopes were clarified in the context of the women’s challenging situations. In describing their hopes, the women were neither overoptimistic or overstated their actual situations, nor caused embarrassment to the healthcare providers. The importance of supporting their involvement in baby matters in the way each family wants also emerged. However, several barriers to fulfilling the women’s hopes were identified including the babies’ conditions and hospital regulations against family visits or presence.

**Conclusion:**

All three themes identified in the study provide important insights for analyzing more deeply ways of implementing a family-centered care for women diagnosed with fetal anomalies in perinatal settings. To improve women’s engagement in decision-making as a team member, women’s hopes should be treated with dignity and respect, and included in the perinatal care of women with abnormal fetuses. Further research is needed to improve the inclusion of women’s hopes in their care in clinical settings.

**Trial registration:**

UMIN Clinical Trials Registry: UMIN000033622 (First registration date: 03/08/2018).

**Supplementary Information:**

The online version contains supplementary material available at 10.1186/s12884-022-05115-x.

## Background

Recent advances in prenatal screening and diagnosis have resulted in an increasing number of women receiving a diagnosis of fetal anomalies in pregnancy [1–3]. Prenatal screening and diagnosis provide information not only about fetal conditions that can be treated by medical or surgical interventions, but also about fetal conditions that are difficult to cure or that leave fetuses with serious functional disabilities even if they survive. These conditions are indicated in the classification of fetal anomalies according to severity and their significant diagnostic or prognostic ambiguity by Kaasen et al. [4]. These circumstances have resulted in complicated situations of perinatal settings among women, their families, and healthcare providers. Thus, a pressing concern is identifying the assistance that can be provided to these women and their families.

Family-Centered Care (FCC) has been consistently promoted as the gold standard approach to providing care in the perinatal and neonatal periods [5]. Similarly in Japan, it has been increasingly recognized that the family should be pivotal in providing care, particularly neonatal and pediatric care [6]. Different FCC-based approaches with a Western impact are now being implemented in Japan [7]. FCC is an approach to care rather than a care model that is uniformly implemented in all clinical settings. Different interventions and care models that are focused on various FCC components have been developed in various settings [8–10]. A scoping review of 55 studies of FCC models for diverse diseases and age groups [10] has revealed that a consistent goal across all the FCC models was to develop and implement a patient care plan within the context of families. This goal is similar to the single goal in perinatal palliative care.

Perinatal palliative care is a relatively new and developing field for women whose fetus or baby is diagnosed with a life-limiting condition [11, 12]. The development of Birth Plans (BPs), which is a critical component of perinatal palliative care and used similarly to Advance Care Planning (ACP), reflects the exploration of goals of care, summarizes the pregnancy journey, and shares the voice of the family while simultaneously documenting requests to all healthcare providers to ensure appropriate care during labor, delivery, and the neonatal period for both the mother and the baby [13]. It enables a family to participate in decision-making and regain a sense of control [13–16].

The lead author (MKC) developed and evaluated a new program mainly focused on supporting the creation of BPs for women diagnosed with fetal anomalies by applying the concepts of birth planning and ACP as practiced in perinatal palliative care [17]. The results suggest that supporting the creation of BPs paves the way to providing guidance to healthcare providers in effectively implementing FCC.

Several studies have shown the negative attitudes of healthcare providers toward BPs, resulting in a negative impact on obstetric/neonatal outcomes, unrealistic expectations, or a false sense of control over the birth [18–21]. The use of BPs may also add challenges to healthcare providers in caring for women diagnosed with fetal anomalies. Some clinicians may hesitate to spell out a plan in situations of uncertainty [3]. Cortezzo et al. [14] also revealed that more than one-third of the physicians who participated in their study did not have sufficient time to complete the BPs with parents, and that some of the physicians felt uncomfortable creating the BPs with the family. A lack of education and training of healthcare providers on caring for critically ill babies was also reported [22, 23].

Documenting women’s hopes expressed in BPs is essential for healthcare providers to ably facilitate women to facilitate women’s care. Therefore, we aimed to clarify in this study the hopes of women concerning birth and parenting after being diagnosed with fetal anomalies.

In this study, we defined BPs as “hopes for pregnancy, childbirth, and the postpartum period, including various ‘hope’-like concepts such as preferences, desires, expectations, views, values, and wishes.” This definition is based on two points: 1) BPs are explained using the same concepts of hope in previous studies of BPs, and 2) BPs include two aspects of hope as defined by Dufault et al. [24], namely, “particularized hope” which is concrete and tangible toward one’s hopes, and “generalized hope” which is vague and broad in scope and not linked to any particular concrete or objective hope. BPs cover a wide range of aspects from pregnancy to postpartum in the context of perinatal palliative care. We defined a BP form as a document created to communicate the BPs to the care team.

## Methods

### Study design

We performed a descriptive qualitative study. The paradigm of the study was pragmatism. Pragmatism allows researchers to be sufficiently flexible to adopt the most practical approach to answer research questions [25]. We believe that it is necessary to build knowledge and transform care through flexible methodologies. This will enable the implementation of care based on the hopes of women diagnosed with fetal abnormalities. The recommended guideline, Standards for Reporting Qualitative Research (SRQR) [26] was used for reporting this descriptive qualitative study.

### Study setting and participants

The study setting was a tertiary perinatal medical center in a metropolitan area of Japan which specializes in fetal diagnosis and management. The center has an average of 450 deliveries per year. It has an obstetrician-attending physician system from the first visit. Prenatal visit by neonatologists was performed if the fetus was diagnosed with abnormalities. We selected this center as it specializes in neonatal and pediatric care, and it was a place where many women with suspected fetal abnormalities around the area were referred to.

We recruited women diagnosed with fetal anomalies and who met the following inclusion criteria: more than 22 weeks of gestation; over 20 years old; able to communicate, read, and write in Japanese; scheduled their childbirth at the study site; recruited between April 2019 and September 2019. The study was explained to eligible women both in writing and verbally by the lead researcher (MKC) or an outpatient department certified-nurse midwife (CNM) with more than 30 years of experience. If a woman was willing to participate, she and her husband signed an informed consent form. A refusal form was also provided to the eligible woman explaining that she can stop participating in the study at any time.

The participants were given a BP form and provided support for creating their BPs by outpatient midwives in more than two meetings. They were asked to submit their BP form by the time of onset of labor. The BP form consisted of the following four open-ended questions: “How do you feel about the current pregnancy and childbirth, or your baby?”, “What do you want to know?”, “How do you want to spend your childbirth?”, and “How do you spend time with your child and what do you want your child to do?”

### Data collection and analysis

Data were collected from the BP forms submitted by the women between April 2019 and December 2019. To clarify the contents of the women’s hopes by identifying the constructs within the text using words or sentences written on their BP forms, the data were analyzed using qualitative content analysis in the following steps in accordance with Graneheim and Lundman [27].

Initially, each description of the women’s BP forms was anonymized and digitized, and was carefully read several times to understand their whole feelings and situations as a unit of analysis. Then, these descriptions were divided into meaning units, which were sentences or words that contain a single answer to the question “What did the women describe as their hopes in the BP form?”. When there were multiple answers in a single sentence, each of these answers was treated as a separate meaning unit. These descriptions included the data added by the CNM through discussions with the women. Considering their contexts, the meaning units were condensed into a description close to the text (the manifest content), where possible, into an interpretation of the underlying meaning (the latent content), and created as codes. According to their commonality, the codes were abstracted into subcategories, and these subcategories were then abstracted into categories and themes.

To ensure credibility, we used data triangulation and researcher triangulation [28]. Data from field notes, memos, and electric medical charts were used. All analyses were performed initially by the first author (MKC) who has experience working as a CNM for over 10 years and helping women to create BP forms. Analysis of one-third of the data was independently performed by a co-author (SI) who has experience caring for women with high-risk pregnancy as a CNM, as well as providing care in a Neonatal Intensive Care Unit (NICU). Similarities and differences were discussed openly within the research team, and a detailed description of the research process was presented and then confirmed by all authors. An example of the procedure is shown in Additional file 1.

Demographic data of the participants were also collected from the electrical medical chart and analyzed descriptively.

### Ethical consideration

This study was conducted in accordance with the Declaration of Helsinki [29] and Ethical Guidelines for Medical and Health Research Involving Human Subjects [30]. This study was performed with the approval of St. Luke’s International University Research Ethics Review Committee [approval no: 18-A035] and Kanagawa Children’s Medical Center Ethics Committee [approval no: 111–10]. It was previously registered in the Clinical Trials Registry of University Hospital Medical Information Network in Japan [UMIN000033622 (The first registration date: 03/08/2018)]. Ethical consideration was carefully paid as the study participants were in the middle of experiencing a loss. All participants and their partners provided written informed consent to participate.

## Results

The BP forms submitted by the 24 women were included in the analysis. The women’s demographics are summarized in Table 1 as follows: their mean age was 33.8 years [SD 4.98]; the number of primiparas was 5 (20.8%); the number of multiparas was 19 (79.2%); all had singletons; and all were married. Congenital heart disease (CHD) was the most common diagnosis among the fetuses of the study participants. All fetuses were anticipated to likely require early postnatal surgery, intensive care in the NICU, or perinatal palliative care.

**Table 1:** Demographics of participants

		*N* = 24
Variable	n (%) / mean [SD]	
Age (range 24–43) years	33.8	[4.98]
20–24	1	(4.2)
25–29	4	(16.7)
30–34	7	(29.2)
35–39	8	(33.3)
More than 40	4	(16.7)
Parity		
Primipara	5	(20.8)
Multipara	19	(79.2)
Number of fetuses		
Singleton	24	(100.0)
Multiple	0	(0.0)
Marital status		
Yes	24	(100.0)
No	0	(0.0)
Fetal diagnoses		
Congenital heart disease	10	(41.7)
Congenital diaphragmatic hernia	3	(12.5)
Craniomaxillofacial disorders	3	(12.5)
Neural tube defects	2	(8.3)
Gastrointestinal obstruction	2	(8.3)
Others	4	(16.7)
Gestational age at the time of the first visit to the research facility (weeks, range 20.6–34.7)	28.7	[4.02]
The time of the beginning of support for BP creation (weeks, range 25.4–37.7)	32.9	[2.94]
Gestational age at giving birth (weeks, range 37.0–41.0)	39.0	[1.05]
The time between the first visit to the research facility and giving birth (weeks, range 4.7–19.0)	10.2	[4.00]
The time between the beginning of support for BP creation and giving birth (weeks, range 1.6–12.9)	6.0	[2.87]

The women visited the research site for the first time at 28.7 gestational weeks [SD 4.02], and they gave birth at 39.0 gestational weeks [SD 1.05]. The average time between the first visit to the research facility and giving birth was 10.2 weeks [range 4.7–19.0, SD 4.00]. The time between the beginning of support for BP creation and giving birth was 6.0 weeks [range 1.6–12.9, SD 2.87].

The analysis of data identified three themes: Hopes as women who are expecting childbirth; Hopes as mothers of a baby; Hopes of being involved in the family needs. These themes consisted of six categories and 19 subcategories (Table 2). Some women’s BP forms included descriptions by their husbands. All of them were analyzed as women’s hopes because all BPs were presented by the women. The results were supported with direct texts from the women’s BPs, and each of the BP forms provided the age, number of the baby, and fetal diagnosis as a background.

**Table 2:** Themes, categories, subcategories, and codes of women’s hopes

Theme/Category (n)	Subcategory	Code
**Theme 1: Hopes as women who are expecting childbirth**		
Preferences during labor and giving birth	Preferences for spending time during labor and delivery	Playing music or video
		Trying to go into childbirth while relaxing
		Providing pain relief
		Adjusting to an environment
		Giving support for overcoming labor and birth
		Encouraging active movements during the first stage of labor
		Using aromatic oils
	Companionship	The husband’s presence
		Fear of being alone
		Preference for husband not to be present
		Own mother’s presence
	Meeting and touching the baby just after giving birth	Early skin-to-skin contact
		Touching the baby
	Mode of delivery and medical interventions	Vaginal delivery
		Minimal medical intervention
	Being well-informed during labor and giving birth	Seeing the placenta
		Knowing the progress or prospect of giving birth
		Knowing if any medical intervention is required
Request for maternal care	Maternal physical needs in postpartum	Putting on a pelvic belt after childbirth
		Taking enough rest after giving birth
		Caring for postpartum urinary incontinence
		Controlling post-cesarean section pain
	Request for maternal treatment plans	Refraining from having aminioreduction
		Giving priority to the mother when a decision is required regarding fetal or maternal treatment
		Aiming for early discharge after childbirth
	Words that participants do not want medical professionals to use	Words that participants do not want medical professionals to use
**Theme 2: Hopes as mothers of a baby**		
Parental roles after giving birth	Spending time and being around the baby	Spending time with and being around the baby
		Holding the baby
		Staying with the baby in the same room
		Enjoying the time alone with the baby
		Spending time with the baby and keeping my pace
	Doing daily care	Breastfeeding
		Doing everything I can do for the baby
		Bathing the baby
		Changing the diapers
		Clipping the baby’s nails
	Memory-making	Taking the baby’s photo just after being born
		Shooting a family photo with the baby just after being born
		Making hand/footprints
	Understanding the baby’s condition and treatments	Knowing the baby’s examination results and condition
		Understanding how to cope with the baby’s symptoms after discharge
Baby’s safety and comfort	Being born safely with strength	Born safely
		Born alive
		Born strong
	Overcoming illnesses and treatments	Hoping that the diseased part of my baby will heal well
		Wishing to get through the treatment and then go home
	Requesting for the baby’s care or treatments	Making their baby as the first priority
		Entrusting healthcare professionals regarding their baby’s treatments
		Making a final decision on the baby’s treatments after childbirth
		Refraining from taking life-prolonging treatments that are unreasonable for the baby
**Theme 3: Hopes of being involved in the family needs**		
Respect family members to be involved in the baby’s matters	Ensuring family time	Family visits
		Spending time with family
	Supporting family members in welcoming the baby	Supporting the husband to participate in giving birth
		Supporting the families to participate in welcoming the baby
Disengagement after the baby is born	Resolving not to think about anything right now	Trying not to think about it too much
	Opting not to know everything before the baby is born	Refraining from knowing everything before the baby is born

### Theme 1: hopes as women who are expecting childbirth

The women expressed concretely what they hope their birth would be like and how they would like to receive support from their surroundings as women who are expecting childbirth. This theme consisted of two categories as follows: Preferences during labor and giving birth and Request for maternal care.

#### Preferences during labor and giving birth

The women mentioned their concrete needs for getting through their childbirth along with their babies with whom they had spent their pregnancy time. Many of them mentioned their specific preferences for spending time during labor and delivery as a way to relief the tension in their body and mind from childbirth, as shown in Table 2. Some expressed their enthusiasm in trying to have childbirth while relaxing as they were very aware that their childbirth had more risks than others.

“I want to give birth with peace of mind, knowing that I will be treated at a specialized hospital, despite the severity of my situation.” (35 yrs. old, second child, CHD).

Many of the women hoped that their husbands would be present during their labor as a companion. If the husband was not available depending on the situation of each woman, they hoped to rely on a CNM or their own mothers to ensure that they would not be alone.

“I would like to have someone from the hospital staff with me, even only when they are available. It would be very encouraging.” (27 yrs. old, fifth child, congenital diaphragmatic hernia).

Additionally, many of them mentioned the need for early skin-to-skin contact, as they had been informed that early skin-to-skin contact was usually available with vaginal deliveries and the neonatologist’s permission. They understood that this may not be possible if they switch to emergency cesarean section to ensure the baby’s optimal condition to start treatment, thus they imagined to interact with their baby in a way that was adapted to their situation.

“I want to touch my baby! If I must have a C-section due to abnormal rotation, I still hope some cheek-to-cheek contact at least!” (35 yrs. old, second child, craniomaxillofacial disorders).

“I know it is difficult because I am currently scheduled for a cesarean section due to a breech baby, but I would like to be able to have a near form of early skin-to-skin contact if possible.” (33 yrs. old, second child, craniomaxillofacial disorders).

Some women mentioned their preferences regarding the mode of delivery or medical interventions. However, they understood that more extensive medical intervention was occasionally required to complete the childbirth safely.

“I hope for a natural birth, but I want an episiotomy or other necessary treatment to ensure a safe birth.” (29 yr old, first child, CHD).

#### Request for maternal care

The women hoped that their own physical and emotional needs would be fulfilled by their healthcare providers, and that they would be able to participate in the decision-making process of their treatment which would directly involve their bodies.

Some women specifically mentioned their various maternal physical needs, such as putting on a pelvic belt after childbirth, taking sufficient rest after giving birth, and controlling post-cesarean section pain. A woman was concerned about postpartum urinary incontinence and she mentioned,

“During my last delivery, I had trouble controlling my urination for a month and often had to use the shower after using the restroom during the hospital stay. If I have the same symptoms this time, and if the hospital allows it, I would like a private room.” (40 yrs. old, second child, CHD).

A few of the women mentioned their request regarding their own treatment plan. For example, a woman was required to undergo the more invasive cesarean section than a normal delivery to save her baby’s life. However, the baby’s prognosis was still uncertain even if the more invasive cesarean section was performed.

“If there is to be a choice between me and my child, I prefer to be the priority because I still have to work hard as a mother and provide for my older child.” (43 yrs. old, second child, congenital vascular malformation).

Sometimes what other people said out of good will made one of the women uncomfortable. She, who was a medical professional on her baby’s diseases, knew firsthand the hardships and suffering the mothers had to go through with these treatments. She mentioned,

“Other people including medical professionals quite often say, ‘your baby chose you’ or ‘the surgery will make it beautiful, but I don’t appreciate it any more”. (33 yrs. old, second child, craniomaxillofacial disorders).

### Theme 2: hopes as mothers of a baby

The women expressed their hopes from the perspective of being responsible for their baby and caring for them with attachment and compassion, being the mothers of their babies. This theme consisted of two categories: Parental roles after giving birth and Baby’s safety and comfort.

#### Parental roles after giving birth

The women mentioned their hopes of trying to carry out their parental roles with responsibility as a mother and their concerted attempts to develop attachment to their babies after giving birth in a situation wherein they anticipated being separated from their babies sooner after giving birth.

Most of the women hoped to be with and around their babies as much as possible. They expected to hold their babies, spend time in the same room or at their own pace, and enjoy their time together,

“As long as my energy lasts, I hope to stay around my baby.” (41 yrs. old, first child, CHD).

“It would be wonderful if I can spend [time] with my baby every day, even for a short time.” (35 yrs. old, second child, CHD).

Medical treatments were required for their babies, which made it uncertain for some women to imagine what they could do after the babies were born. With the help of healthcare providers, they tried to imagine providing daily care which could be done in the baby’s situation such as pumping breast milk, changing diapers, bathing, or clipping nails.

They also mentioned memory-making, such as taking photos, shooting videos, and making hand/footprints. They especially valued making mementos with their babies just after being born, as their babies would have to be separated sooner after giving birth,

“If it’s OK, I’d like to get pictures after he is born in the same pose as when my older daughters were born.” (29 yrs. old, third child, gastrointestinal obstruction).

In addition, some women mentioned their hopes to understand their baby’s examination or condition and how to cope with their symptoms after they leave the hospital as mothers. Some women occasionally expressed their feelings of blaming themselves regarding their BPs as they contemplated their parental roles,

“I do feel terribly sorry for her and about her illness as well. Because she is a girl … … … I hope to do everything I can do for her.” (26 yrs. old, second child, CHD).

#### Baby’s safety and comfort

Childbirth was anticipated to be the first and greatest stress in their babies’ life. The women who could not avoid being separated from their babies after childbirth or who faced their baby’s end-of-life care mentioned their hope and prayer that they were eager for their babies to be safe and comfortable.

The women hoped for their babies to be born safely, alive, and with the strength to be able to overcome some painful treatments.

“I know I will put you through a lot of hardship right after you are born. I’m sorry..., but I want you to be born strong.” (43 yrs. old, first child, congenital diaphragmatic hernia).

Particularly, it was hard for the women to think about their babies’ birth and death separately if the healthcare providers deemed that the baby would have a life-limiting condition.

“ALL I hope is to see my baby alive and trying to live. If she can be born alive, I won’t expect any more.” (32 yrs. old, first child, CHD).

Many of the women mentioned the importance of making their baby’s condition or forming a medical judgment regarding their baby’s care and treatments as their priority, for example,

“I hope the baby’s situation takes first priority over all [other] hopes.” (40 yrs. old, second child, CHD).

“I entrust healthcare professionals regarding my baby’s treatments.” (29 yrs. old, first child, CHD).

In some cases, the treatment plan could not be determined in advance owing to the greater uncertainty of the baby’s prognosis. Some women had been informed that their babies might still be in severe condition even with intensive care. The women expressed their intentions regarding such conditions based on their own values.

“If my baby requires a tracheotomy before surgical intervention, no further life-prolonging treatment is desired.” (43 yrs. old, second child, congenital vascular malformation).

“I strongly ask the medical team to make sure that she is REALLY untreatable after she is born.” (32 yrs. old, first child, CHD).

### Theme 3. Hopes of being involved in the family needs

The women hoped that their healthcare providers would also allow their families to participate in matters concerning the babies as they needed. This theme consisted of two categories: Respect for family members to be involved in the baby’s matters and Disengagement after the baby is born.

#### Respect family members to be involved in the baby’s matters

The women mentioned their hopes that they would like to be ensured family time, and that family members (i.e., husbands, baby’s grandparents, older children) who were looking forward to welcoming the baby would receive support for being involved in their baby’s matters together with the healthcare providers, despite the inevitable limited access to their newborns after giving birth.

The birth of their baby provides enormous relief to women from their great stress and marks a precious time for all the family members to welcome a new family member. Many women hoped to be ensured of family time to be able to spend time with close family members.

“I JUST want to have some family time.” (35 yrs. old, second child, chromosomal abnormality).

In addition, the women hoped that their family members would receive support in welcoming the baby, especially the participation of their husbands in their childbirth, for example, telling them how to massage or letting them hold the baby.

“When she is born, I want my husband to hold her first because I have felt her movement every day, but he could not.” (32 yrs. old, first child, CHD).

Some hopes were not easy to fulfil under the baby’s situation or hospital regulations. Most of the women often mentioned that they wanted their older children to meet the new family member, whereas some women wanted their baby’s uncle/aunt to meet the new family member. In addition, as a regulation in the study site, explanations regarding the baby’s condition were provided only to the parents by healthcare providers as a form of respect for the parents own decision-making. For example, a woman mentioned,

“When a meeting is held to explain the details of the disease [of my baby], we fervently hope that the baby’s grandparents will be allowed to stay with us. If the parents express this hope greatly, I want the healthcare providers to allow it.” (37 yrs. old, first child, congenital diaphragmatic hernia).

These hopes required advanced discussion and coordination with a multidisciplinary team. Furthermore, a woman whose baby was planned to be intubated immediately after birth mentioned, “I want my parents to see my baby first before they attach any medical device on him” (37 yr old, first child, congenital diaphragmatic hernia). Another woman who anticipated that her baby’s life was limited after birth mentioned, “Let my baby be with all our family members, siblings, and parents while she is alive, if possible.” (32 yr old, first child, CHD). These hopes were unfortunately not realizable under the baby’s situation or hospital regulations, and an additional meeting was held to explain to the women the difficulties in fulfilling these hopes. The women understood the circumstances and accepted more realistic alternatives.

#### Disengagement after the baby is born

Some women mentioned their intention to refrain from thinking or knowing about their present and future hopes. These feelings were expressed by women whose babies were diagnosed with a life-threatening condition, or by women with a deep understanding of the painful treatment process that their babies would go through.

“I don’t want to know so much things before our baby is born.” (35 yrs. old, second child, chromosomal abnormality).

“Now I want to enjoy the time I have with my family (husband and son) without thinking about anything.” (33 yrs. old, second child, craniomaxillofacial disorders).

## Discussion

In this study, we set out to clarify the hopes of women regarding birth and parenting after being diagnosed with fetal anomalies. From the women’s hopes, three themes emerged: Hopes as women who are expecting childbirth, Hopes as mothers of a baby, and Hopes of being involved in the family needs. The present results provide a better understanding of these women’s BPs and their hopes. These hopes provide important information for analyzing more deeply ways of implementing an FCC for women diagnosed with fetal anomalies in perinatal settings.

Some of the contents of the hopes of the pregnant women diagnosed with fetal anomalies in the present study showed similarities with the contents of the hopes of pregnant women without risks of fetal anomalies, as cited in the web pages providing guidance on how to create BPs for pregnant women [31, 32] and in the NICE guidelines [33]. The first theme (Hopes as women who are expecting childbirth) expressed what women would like to happen during their childbirth and after giving birth, even though they had been diagnosed with fetal anomalies. These hopes appeared to be natural for the women who were going to give birth.

On the other hand, many distinctive hopes were also identified. One of the categories of the second theme (Hopes as mothers of a baby), that is, Parental roles after giving birth, was mentioned concretely by most of the women. Some women fervently hoped to do everything for their babies as mothers because they blamed themselves for their babies’ anomalies. It appeared that the women had distinctive hopes in that they were willing to fulfill their parental roles in their own ways despite the inevitable separation with the babies.

More distinctive was the fact that many women mentioned vague hopes with an uncertain future, such as the subcategories “Being born safety with strength” and “Overcoming illnesses and treatments”, as indicated under the second category Baby’s safety and comfort. These hopes appeared to represent “generalized hope”, one of the two spheres of hope defined by Default et al. [24], as opposed to “particularized hope” that is tangible and realizable in the future. Default et al. described that generalized hope is “like an intangible umbrella that protects a person with hope by casting a positive glow on life” [24]. In the present study, desperate hopes were manifested in prayers by women who could not avoid being separated from their baby or who faced their baby’s end-of-life care.

In addition, our findings showed that the women’s hopes were not overoptimistic or overexpectations beyond reality, and that these hopes were not intended to cause any embarrassment to the healthcare providers. Several studies have revealed the reasons why healthcare providers had negative attitudes toward BPs. In a qualitative study of nine maternity midwives, Welsh and Symon [21] have indicated that the term “birth plans” could be misleading and may contribute to the women’s unrealistic expectations during labor. Aragon et al. [18] have also revealed that BPs caused a wrong sense of control over childbirth. Furthermore, the non-fulfillment of their expectations causes dissatisfaction among women [18, 34], which is another aspect that healthcare providers view as negative regarding BPs.

In the present study, the context of each woman was taken into account by discussion with and suggestions from the midwives as support in the creation of BPs, making the women’s BPs individualized and tailor-made. This approach fulfilled most of the hopes of the women except for vague hopes such as baby’s safety and comfort which was classified as generalized hope. If the hopes required other explanations or prior coordination within a multidisciplinary team because of the baby’s situation or hospital regulations, the women understood the situation and accepted the alternative with additional explanations by the healthcare providers, as seen in the third theme, Hopes of being involved in family’s needs. Moreover, the women in the present study actually prioritized their babies and their own medical conditions over any of their hopes, as mentioned in the first and second themes. In other words, the women did not consider their BPs as being strict, but as flexible guidelines for their situations.

Wilpers et al. [35] reported that women’s hopes amidst uncertainty or a poor prognosis could easily be misinterpreted as optimistic when healthcare providers are unaware of the unique hopes of the parents. Thus, it would be possible to avoid the negative views of BPs by clinicians even in obstetric-led situations in which fetal anomalies are diagnosed if healthcare providers understand the unique hopes of women and if it is possible to discuss these hopes between the woman and the healthcare provider in advance. We believe that the women’s hopes identified in the present study would be helpful in developing an educational program for healthcare providers on the effective implementation of FCC in the perinatal period. To improve the engagement of women in the baby’s care as a team member, it is also necessary to identify facilitators and barriers to the inclusion of women’s BPs in clinical care.

Furthermore, our findings show the importance of considering each family’s situations and needs, and the value of supporting their involvement in the baby’s matters according to the needs and wants of each family. In the third theme, Hopes of being involved in the family needs, most of the women hoped that their family members could participate and receive support in welcoming the baby. The women fervently hoped that the time with all family members would be ensured and considered precious by each family member. Nevertheless, there were several barriers to fulfilling their hopes such as their babies’ conditions and hospital regulations against family visits.

In their integrative review of 44 studies based on the perspectives of parents who had experienced care in a pediatric intensive care unit [9], Hill et al. found that parents often reported environmental- and healthcare provider-related barriers to participating in their child’s care at the level they preferred. These levels may vary from how long parents were permitted to stay in the hospital or whether they receive help from the care providers participating in their baby’s care. In a cross-sectional survey of 52 NICUs in Japan, which is equivalent to about half of Japan’s tertiary NICUs [36], Ozawa et al. reported that less than half of these NICUs allowed the parents to be present for 24 hours at the NICU before the coronavirus disease pandemic. They also described that the NICU policy restricted the number of family members and the family member who could visit the NICU. These were the common barriers to the participation of parents and their family to the baby’s care.

On the other hand, some women mentioned that they did not want to engage or dwell on the matter at this time, as indicated in the second category of the third theme, Disengagement after the baby is born. This indicates that there are individual needs in expressing hopes of the women and their families regarding the care they receive.

The creation of BPs in painful and challenging situations would have forced the women to imagine a future in which their babies would be born with diseases or will be dying shortly. BP creation in this situation would have a similar meaning to ACP as used in the context of perinatal palliative care. The NICE guidelines of end-of-life care for infants, children, and young people with life-limiting conditions [37] have noted that discussing ACP could be painful for terminally ill children and youth as well as their parents and caregivers. Similarly, Côté-Arsenault and Denney-Koelsch [38] found that some parents gain some control over their situation as they prepare for childbirth and inevitable death through planning. In contrast, other parents are unable to gain such control and are therefore unwilling to plan similarly.

Hence, it is essential that women and their families should be encouraged and supported to participate in the care and decision-making of their new baby at the levels they deem appropriate, as defined by the Institute for Patient- and Family-Centered Care [39]. Discussing and clarifying how the parents’ hope to participate in the care of their newborn are thought to be increasingly important aspects for implementing FCC in prenatal settings.

### Strengths and limitations

The strength of the present study is that the findings are based on real voices of women diagnosed with fetal anomalies. These real voices and hopes are anticipated to be useful in guiding healthcare professionals or organizations in the effective implementation of FCC in the perinatal period. All these aspects of care and BPs should be treated with respect as the women are coping with or facing unexpected and challenging situations. For the study limitations, the data were obtained only from a single facility and the sampling was unbalanced on parity.

## Conclusions

We identified three themes of hopes from the content analysis of the BPs created by women diagnosed with fetal anomalies. These themes represent a valuable voice of women diagnosed with fetal anomalies and can be useful in guiding healthcare providers in effectively implementing FCC in the perinatal period. Further research is needed to improve the inclusion of these women’s hopes when providing them with care in clinical settings.

## Supplementary Information

**Additional file 1.**

## Data Availability

The datasets generated and/or analyzed in the current study are not publicly available in line with the ethics approval. Data are available from the lead author (MKC) upon reasonable request and subject to approval of the ethics committee.

## Abbreviations

FCC: Family-Centered Care
BPs: Birth Plans
ACP: Advance Care Planning
NICU: Neonatal Intensive Care Unit
CHD: Congenital Heart Disease
CNM: Certified Nurse-Midwife
